# European birth cohorts offer insights on environmental factors affecting human development and health

**DOI:** 10.1093/ije/dyv132

**Published:** 2015-07-29

**Authors:** Katarzyna Kordas, Alison Park

**Affiliations:** Avon Longitudinal Study of Parents and Children (ALSPAC), School of Social and Community Medicine, University of Bristol, Bristol, UK; CLOSER, Institute of Education, University College London, London, UK

European countries, particularly the UK, have a long history of conducting birth cohort studies. Now a new birth cohort, the UK’s Life Study, has ambitious plans to enroll tens of thousands of participants and a hope that it will succeed where its counterpart in the USA, the National Children’s Study (NCS) has failed.^1^ The NCS was dissolved in December 2014 by the Director of the National Institutes of Health (NIH) amid concerns over the study’s feasibility and costs.^2^^,^^3^ Although the NIH remains committed to the overarching goals of the NCS,^2^ to ‘find alternative ways to study child development and environmental influences on health’,^4^ it is unclear when and how this is likely to happen. However, ready options to address the goals of NCS are available among the European birth cohorts^5^^,^^6^ and are likely to have international relevance beyond Europe.

Authorized by the Children’s Health Act of 2000, the NCS was a prospective birth cohort study developed to evaluate the effects of environmental exposures on child health and human development.^2^^,^^7^ The vision for the NCS was to enrol a probability sample of 100 000 children and examine a broad range of environmental and biological factors. The study was meant to be a ‘complete assessment of the physical, chemical, biological and psychosocial environmental influences on children’s well-being’^2^ and to address socioeconomic and racial/ethnic disparities in environmental exposures and health outcomes.^2^ Several Vanguard Centres were launched to test the feasibility, acceptability and costs of running the main study. But after years of struggling to get off the ground and numerous criticisms, a working group set up to review the fate of the study concluded that although the goals of the NCS were meritorious, the study was not feasible in its current form.^2^

The longitudinal birth cohort design was one of the strengths of the NCS when a panel of experts was convened to evaluate the study and recommend its future.^2^ In its initial design, the NCS planned on enrolling women before conception, to account for antenatal exposures. However, given the large costs, slow pace and inefficiencies involved with this recruitment strategy, the traditional recruitment at antenatal visits seemed appropriate and justified^7^ and was ultimately adapted.^8^

Collectively, European birth cohorts offer an excellent opportunity to investigate the types of questions that the NCS was intended to address. These cohorts bring together several key elements, in terms of sample size, sampling strategy, exposure measures and phenotypic richness, to fill the scientific gap left by the closure of the NCS. Some of the larger cohorts have been hailed as exemplars of efficiency in study development and implementation,^7^^,^^8^ but all of them are excellent resources to investigate the potential causal influences of many biological, social and environmental factors on human health. Most importantly, the scientific community does not have to wait over 20 years for the answers. Some of the cohorts already offer life-course and even inter-generational perspectives on human health and can contribute real, including mechanistic, insights into the picture of the health-disease continuum. In addition, many of these studies are working collaboratively to address a range of health issues.^5^^,^^6^^,^^9^

The many European cohorts in existence—descriptions and links to individual study websites at [http://birthcohorts.net/]—are varied in focus, each offering unique features and strengths. Many have taken the approach of enrolling women in pregnancy or the children shortly after birth. One of the exceptions is the Southampton Women’s Survey (SWS) in the UK,^10^ which enrolled women pre-conception. These cohorts range in size from relatively small (SWS) to very large (for example, the Norwegian Mother and Child Cohort Study—MoBa,^11^ the Danish National Birth Cohort—DNBC^12^ and the UK’s Life Study), in their geographical focus from local (SWS, the UK’s Avon Longitudinal Study of Parents and Children, ALSPAC,^13^^,^^14^ The Netherlands’ Generation R study^15^) to nationally representative (Life Study, the UK’s Millennium Cohort Study, MoBa, DNBC), in data collection methods and in the range and depth of their phenotypic characterization (ALSPAC, for example, is one of the most phenotypically rich studies in the world). Many of these studies have sufficiently detailed information to allow major contributions to both biomedical and social science research (for example, ALSPAC and Generation R, among others) and have the potential to contribute to more fundamental biological research. Some of these studies began soon after WWII (the UK’s 1946 and 1958 Birth Cohort Surveys^16^^,^^17^ or as recently as February 2015 (Life Study). They represent a range of exposures to environmental factors, economic conditions and social phenomena. An additional unique feature of some of these cohorts is their linkage to participants’ administrative records (medical, educational, criminal and economic), which not only provides additional outcome measures^18^ but also allows researchers to fill in gaps when missing data or non-response threaten to introduce bias.^19^^,^^20^

European birth cohorts have data and/or samples available for access by the wider scientific community, thereby acting as large repositories of information ready to be interrogated. Precisely because many of the European birth cohorts have collected and archived biological samples, they can be very responsive to the emergence of new technologies, such as sophisticated genetic analyses,^1^ For example, ALSPAC and an increasing number of the other cohorts, are able to prospectively investigate the influences of environmental factors on patterns of DNA methylation in mothers and children^21^^,^^22^ and are currently building metabolomics profiles of their participants across the life cycle. Alternatively, cohorts can use these technological advances to understand how social factors (collected decades earlier) affect the underlying physiological processes.^23^

Clear data access policies are in place in the European cohorts, available data are detailed on publicly accessible websites and decision-making is swift. When finite resources are requested, an independent arbitration process exists to decide whether the proposed study/measures fall within the cohort’s research priorities, are likely to offer novel insights or will yield high-level impact. The studies are governed by small groups of cohort study experts who can respond fairly quickly to the changing scientific and infrastructural needs. Some studies hold public consultations on upcoming waves of surveys or assessments to gather input from the wider scientific community, and thereby keep abreast of trends in a given field of inquiry (the Millennium Cohort Study is one example of this). A real strength of some of the studies (for example, ALSPAC) is the deliberate involvement of cohort participants in research design and governance^24^ and the emphasis on cohort engagement strategy and research.^25^ Infrastructural elements allowing flexibility, responsiveness and ‘consensus-driven science’ are critical to the success of a birth cohort.^1^

Many European birth cohorts coordinate efforts with other longitudinal studies, at either the infrastructural or the scientific level, through joint governance, harmonized data collection or metadata or research consortia. For example, several EU-funded projects (CHICOS, ENRIECO, GA^2^LEN, MeDALL and others) brought together a large number of European birth cohorts to address a variety of health issues.^5^^,^^6^^,^^9^ In another example, the CLOSER network [http://www.closer.ac.uk/] brings together a number of the major UK birth cohorts as well as the UK Household Longitudinal Study (Understanding Society), to encourage interdisciplinary research, facilitate the use of longitudinal data and share resources and expertise.

Whereas existing European birth cohorts offer an excellent opportunity for research on early determinants of human development and health, two important issues need to be acknowledged. First, an argument has been made that new cohort studies are needed to account for the changing landscape of exposures.^1^^,^^26^ Children born today enter an increasingly digitized world, with economic uncertainty and greater income disparities. They are also likely to be more affected by climate change and the greater movement of people around the globe than previous cohorts. Second, studies conducted in any given region or country address specific socio-cultural contexts of the area they represent. For example, the NCS reflected the socio-cultural context of the American population^8^^,^^26^ with a very specific ethnic, racial, urban-rural mix and social experiences. Although exposure to some social phenomena is undeniably culture specific, European cohorts represent a wide range of exposures to a variety of common social, biological and chemical factors influential to health, and this range of exposures encompasses those likely to be experienced elsewhere in the world. In fact, epidemiologists already pool, integrate and quantify health effects through systematic reviews and meta-analyses. In the very least, findings from the existing cohorts can suggest specific hypotheses that can be tested in targeted samples in other contexts. However, studies do not need to be representative of the wider population to show valid relationships between exposure and disease.^27^

The European birth cohorts have made important contributions to science and the public health agenda. It is impossible to summarize those here (but see references 28–34 for a few recent examples, showcasing some of the scientific contributions in the areas of prenatal exposures, obesity, respiratory health and social inequalities). Additionally, the cohorts keep the record of their publications and highlight their impact on public health and policy on their websites. Nevertheless, birth cohorts hold considerable untapped potential. Through innovative approaches, such as cross-cohort comparisons, replication studies or large consortia,^35^ European birth cohorts should be utilized to search for preventable causes of poor growth, development and health because they are likely to yield valid, generalizable ‘truths’ about the determinants or causes of health and disease. There is already overlap among exposure and phenotype measures across the European cohorts,^5^^,^^6^ but the issue of measurement standardization is non-trivial and likely to require a great deal of effort^36^ with respect to existing information, and planning with respect to future data collection. To facilitate harmonization, the existing cohorts need to agree on the collection of key common variables, including their periods of reference and categories, without losing their unique attributes. There also needs to be sustained investment from funders to ensure long-term success for greatest health impact. Another important advantage of the existing European cohorts is that some of them, either through cross-generational (ALSPAC) or life-cycle perspectives (the 1946, 1958 and 1970 Birth cohorts in the UK), can provide important insights into early predictors of the disease process and/or outcome in later adulthood.^37^ These findings could be translated now or in the near future into preventative measures for the betterment of society.

## References

[1] Pearson H Wanted: 80,000 British babies for massive study. Nature 2015;518:463–64. 25719641 10.1038/518463a

[2] NIH. National Institutes of Health Advisory Committee to the Director National Children's Study (NCS) Working Group, Final Report. Washington, DC: National Institutes of Health, National Children's Study (NCS) Working Group, 2014.

[3] NRC., IOM. The National Children's Study Research Plan: A review. Panel to review the National Children's Study Research Plan. Washington DC: National Research Council and Institute of Medicine, 2008.

[4] Reardon S NIH ends longitudinal children's study. Nature News and Comment 2014, 12 December.

[5] Larsen PS Kamper-Jørgensen M Adamson A Pregnancy and birth cohort resources in Europe: a large opportunity for aetiological child health research. Paediatr Perinat Epidemiol 2013;27:393–414. 23772942 10.1111/ppe.12060

[6] Vrijheid M Casas M Bergström A European birth cohorts for environmental health research. Environ Health Perspect 2012;120:29–37. 21878421 10.1289/ehp.1103823 PMC3261945

[7] Savitz DA Ness RB Saving the National Children's Study. Epidemiology 2010;21:598–601. 20631622 10.1097/EDE.0b013e3181e942cc

[8] Anonymous. Misplaced childhood. Nature 2012;485:279. 10.1038/485279a 22596113

[9] Bousquet J Anto J Sunyer J Pooling birth cohorts in allergy and asthma: European Union-funded initiatives - a MeDALL, CHICOS, ENRIECO, and GA^2^LEN joint paper. Int Arch Allergy Immunol 2013;161:1–10. 10.1159/000343018 23258290

[10] Inskip HM Godfrey KM Robinson SM Cohort profile: The Southampton Women's Survey. Int J Epidemiol 2006;35:42–48. 16195252 10.1093/ije/dyi202 PMC4579566

[11] Magnus P Irgens LM Haug K Cohort profile: The Norwegian Mother and Child Cohort Study (MoBa). Int J Epidemiol 2006;35:1146–50. 16926217 10.1093/ije/dyl170

[12] Olsen J Melbye M Olsen SF The Danish National Birth Cohort – its background, structure and aim. Scand J Public Health 2001;29:300–07. 11775787 10.1177/14034948010290040201

[13] Boyd A Golding J Macleod J Cohort Profile: The ‘Children of the 90s’—the index offspring of the Avon Longitudinal Study of Parents and Children. Int J Epidemiol 2013;42:111–27. 22507743 10.1093/ije/dys064 PMC3600618

[14] Fraser A Macdonald-Wallis C Tilling K Cohort Profile: The Avon Longitudinal Study of Parents and Children: ALSPAC mothers cohort. Int J Epidemiol 2013;42:97–110. 22507742 10.1093/ije/dys066 PMC3600619

[15] Jaddoe VW van Duijn CM van der Heijden AJ The Generation R Study: design and cohort update until the age of 4 years. Eur J Epidemiol 2008;23:801–11. 19101808 10.1007/s10654-008-9309-4

[16] Power C Elliott J Cohort profile: 1958 British birth cohort (National Child Development Study). Int J Epidemiol 2006;35:34–41. 16155052 10.1093/ije/dyi183

[17] Wadsworth M Kuh D Richards M Hardy R Cohort Profile: The 1946 national Birth Cohort (MRC National Survey of Health and Development). Int J Epidemiol 2006;35:49–54. 16204333 10.1093/ije/dyi201

[18] Cornish RP Boyd A Van Staa T Salisbury C Macleod J Socio-economic position and childhood multimorbidity: a study using linkage between the Avon Longitudinal Study of Parents and Children and the General Practice Research Database. Int J Equity Health 2013;12: 66. 23962118 10.1186/1475-9276-12-66 PMC3751770

[19] Cornish RP Tilling K Boyd A Davies A Macleod J Using linked educational attainment data to reduce bias due to missing outcome data in estimates of the association between the duration of breastfeeding and IQ at 15 years. Int J Epidemiol 2015;44:937–45. 10.1093/ije/dyv035 PMC4521129 25855709

[20] Cornish R Tilling K Boyd A Macleod J Van Staa T Using linkage to electronic primary care records to evaluate recruitment and nonresponse bias in the Avon Longitudinal Study of Parents and Children. Epidemiology 2015;26:e41–42. 25835264 10.1097/EDE.0000000000000288 PMC4454477

[21] Richmond RC Simpkin AJ Woodward G Prenatal exposure to maternal smoking and offspring DNA methylation across the lifecourse: findings from the Avon Longitudinal Study of Parents and Children (ALSPAC). Hum Mol Genet 2015;24:2201–17. 25552657 10.1093/hmg/ddu739 PMC4380069

[22] Sharp GC Lawlor DA Richmond RC Maternal pre-pregnancy BMI and gestational weight gain, offspring DNA methylation and later offspring adiposity: findings from the Avon Longitudinal Study of Parents and Children. Int J Epidemiol 2015;doi: 10.1093/ije/dyv042. 10.1093/ije/dyv042 PMC4588865 25855720

[23] Borghol N Suderman M McArdle W Associations with early-life socio-economic position in adult DNA methylation. Int J Epidemiol 2012;41:62–74. 22422449 10.1093/ije/dyr147 PMC3304522

[24] Kordas K O'Hare D Jacobs-Pearson M Longitudinal studies: Engaged cohort good for science. Nature 2014;516:170. 25503226 10.1038/516170d

[25] Ochieng CA Minion J Turner A Murtagh M Stakeholder views about participating in paediatric biobanks: a narrative review. Int J Hum Soc Sci 2015;2:71–85.

[26] Wadman M Child-study turmoil leaves bitter taste, frustration mounts as ambitious US project is scaled back. Nature 2012;485:287–88. 22596128 10.1038/485287a

[27] Willett WC Merging and emerging cohorts. Nature 2007;445:257–58. 17230171 10.1038/445257a

[28] Magnus MC Håberg SE Karlstad Ø Nafstad P London SJ Nystad W Grandmother's smoking when pregnant with the mother and asthma in the grandchild: the Norwegian Mother and Child Cohort Study. Thorax 2015;70:237–43. 25572596 10.1136/thoraxjnl-2014-206438 PMC5034931

[29] Brandlistuen RE Ystrom E Nulman I Koren G Nordeng H Prenatal paracetamol exposure and child neurodevelopment: a sibling-controlled cohort study. Int J Epidemiol 2013;42:1702–13. 24163279 10.1093/ije/dyt183 PMC3887567

[30] Granell R Henderson AJ Evans DM Effects of BMI, fat mass, and lean mass on asthma in childhood: a Mendelian randomization study. PLoS Med 2014;11:e1001669. 24983943 10.1371/journal.pmed.1001669 PMC4077660

[31] Hansen ML Thulstrup AM Juhl M Kristensen JK Ramlau-Hansen CH Predictors of sickness absence in pregnancy: a Danish cohort study. Scand J Work Environ Health 2015;41:184–93. 25469623 10.5271/sjweh.3470

[32] Maastrup R Hansen BM Kronborg H Breastfeeding progression in preterm infants is influenced by factors in infants, mothers and clinical practice: the results of a national cohort study with high breastfeeding initiation rates. PLoS One 2014;9:e108208. 25251690 10.1371/journal.pone.0108208 PMC4177123

[33] Snelgrove JW Murphy KE Preterm birth and social inequality: assessing the effects of material and psychosocial disadvantage in a UK birth cohort. Acta Obstet Gynecol Scand 2015;94:766–75. 25846179 10.1111/aogs.12648

[34] Johnson W Li L Kuh D Hardy R How has the age-related process of overweight or obesity development changed over time? Co-ordinated analyses of individual participant data from five United Kingdom birth cohorts. PLoS Med 2015;12:e1001828. 25993005 10.1371/journal.pmed.1001828 PMC4437909

[35] Richmond RC Al-Amin A Davey Smith G Relton C Approaches for drawing causal inferences from epidemiological birth cohorts: a review. Early Hum Dev 2014;90:769–80. 25260961 10.1016/j.earlhumdev.2014.08.023 PMC5154380

[36] Collins FS Manolio TA Necessary but not sufficient. Nature 2007;445:259. 17230172 10.1038/445259a

[37] Power C Kuh D Morton S From developmental origins of adult disease to life course research on adult disease: insights from birth cohort studies. Annu Rev Public Health 2013;34:7–28. 23514315 10.1146/annurev-publhealth-031912-114423

